# Study of pig manure digestate pre-treatment for subsequent valorisation by struvite

**DOI:** 10.1007/s11356-020-10918-6

**Published:** 2020-10-03

**Authors:** Francisco Corona, Dolores Hidalgo, Jesús María Martín-Marroquín, Erik Meers

**Affiliations:** 1 CARTIF Centro Tecnológico, 47151, Boecillo, Valladolid, Spain; 2 ITAP Institute, University of Valladolid, 47010 Valladolid, Spain; 3 Department of Applied Analytical and Physical Chemistry, Faculty of Bioscience Engineering, Ghent University, Coupure Links 653, 9000 Ghent, Belgium

**Keywords:** Phosphorus, Acidification, Release, Nutrient recovery, Organic waste, Biofertiliser

## Abstract

This work evaluates the release of phosphorus contained in the digestate from the anaerobic digestion of pig manure, through an acidification process. The objective of this acidification is to increase the amount of phosphorus available in the digestate liquid fraction and, subsequently, recover this element by chemical precipitation in the form of struvite or calcium phosphate. Two digestate samples (one fresh and one old) were studied and treated by adding various amounts of sulphuric acid to the different digestate fractions (raw digestate, solid fraction and liquid fraction). For the raw digestate, phosphorus releases higher than 95% were obtained for pH 4.0. In the last part of the experiment, the influence of acid pre-treatment on the reaction yield of phosphorus precipitation, in the form of struvite or calcium phosphate, was determined. Improvements in reaction yield were obtained up to 15% for struvite and 80% for calcium phosphate, increasing also in 7.5 times the amount of phosphorus available in the digestate liquid fraction, for both cases.

## Introduction

In 2050, the world will have to feed about 9 billion people (Manning 2015). By then, agriculture and livestock could account for an estimated one-third of EU emissions, three times what it currently represents.

Agriculture is one of the most important economic sectors in Europe. Its production value in 2018 was around €434 billion. The production value of livestock represented almost 40% of total agricultural production (€172 billion), highlighting the socio-economic relevance of the sector (Eurostat 2019). Intensive livestock farming is an important source of greenhouse gas (GHG) emissions: mainly methane (CH_4_) and nitrous oxide (N_2_O), derived from a multitude of microbial reactions. Their relative importance depends on the composition of the manure (organic waste), the time and conditions of storage, the treatments applied and the application to the field and the climatic conditions of each scenario. Therefore, with these perspectives, the growth of the sector will in turn cause an increase in its weight in terms of climate policy, since, if emissions do not evolve in the sector according to the EU objectives, other sectors will have to compensate them even more, which would have high costs. The challenge of reducing the environmental and economic impact of manure management will become increasingly important and decisive.

There is currently a conflict between efforts to improve food production and rural development and efforts to reduce GHG emissions from manure. In the transition from an economy based on fossil resources to one based on bioeconomics, the efficient recovery of valuable nutrients from organic waste has become a major challenge. Non-renewable natural nutrient resources such as phosphorous rock, oil or natural gas are rapidly depleting. Significant amounts of fossil energy are used for the production of chemical fertilisers, resulting in considerable impacts related to extraction, manufacture and use (ten Hoeve et al. 2014), while energy and fertiliser prices increase. Several authors have studied the current state of the natural reserves of phosphorous from mineral origin and the estimation of their depletion. According to Van Vuuren et al. (2010), one of the first estimates in this respect came from the Institute of Ecology in 1972. The conclusions of this study highlight the possibility that phosphoric reserves may be exhausted before the end of this century. Steén (2004) shows even worse scenarios where in 2050 half of the phosphorus reserves would be depleted. Although different authors explain that the real reserves of phosphate rocks are not known, practically all claim that between 50 and 150 years the resources will be exhausted (Van Vuuren et al. 2010).

On the other hand, manure has a high potential as an organic fertiliser in agriculture, thanks to its content of nitrogen, phosphorus, potassium and organic matter, among others. Manure has a high content of nutrients, although it depends largely on the type and origin of the manure. It can contain 1500–4000 mgN/L and 500–2000 mgP/L for pig manure or 900–950 mgN/L and 1000–1010 mgP/L for dairy manure (Cai et al. 2013; Fangueiro et al. 2016; Ma et al. 2018). On the other hand, wastewater also has an interesting concentration of nutrients available for recovery, although this concentration is usually considerably lower than in the case of manure, 15–90 mgN/L and 5–20 mgP/L for sewage, 10–500 mgN/L and 10–180 mgP/L for industrial wastewater (Cai et al. 2013). However, the great intensification that livestock has experienced in recent decades has generated the concentration of large amounts of manure in very specific areas, making it difficult to manage. This imbalance, combined with bad practices in manure management in some cases, is one of the most worrying aspects of public opinion. Manure imbalance can cause environmental problems, such as pollution by GHG and ammonia emissions, nitrate filtration to groundwater, eutrophication of surface water, accumulation of metals and phosphorus in soils and spread of pathogens, not to mention social rejection produced by bad odours.

According to Foged et al. (2012), the combined population of pigs, cattle, poultry, sheep and goats produces more than 1400 Mt of manure per year. This means that, due to livestock droppings, an estimated 7–9 Mt of nitrogen and 1.8 Mt of phosphorus (in the form of livestock waste) are available each year in Europe. Thus, both organic matter and phosphorus from livestock waste are resources with great potential and a valuable interest in their recovery and reuse. Moreover, this is a priority issue, bearing in mind that mineral phosphorus is a non-renewable raw material and does not have a substitute product. However, there is an imbalance in the phosphorus cycle as far as the European agricultural sector is concerned. Furthermore, it is very important to consider that the current legislation is very restrictive with regard to the direct use of livestock residues on crops. Largely, this is due to their concentration of phosphorus and nitrogen (indiscriminate fertilisation of these nutrients can cause serious environmental damage) and in addition, these restrictions will be increasingly severe. Therefore, the need to process these wastes in an appropriate way is urgent.

Work is currently underway on legislation at European level to harmonise the use of livestock waste and the associated nutrients. The new EU Fertiliser Regulation will include the use of recovered biofertilisers. The revision of the EU Fertiliser Regulation is progressing and the proposed legislation is currently being negotiated by the European Parliament, the Council of the European Union and the European Commission. Therefore, it is necessary to encourage the development and implementation of techniques to recover the nutrients available in agricultural waste. Phosphorus can be recovered both from the manure itself and from the by-products (digestate) obtained in the anaerobic digestion (AD) of these wastes. According to Schoumans et al. (2015), there are several ways to recover nutrients from livestock waste. The technology to be used depends on the fraction (solid, liquid or raw) from which the nutrients are to be recovered, as well as the final product to be obtained (biofertiliser).

One of the most promising methods of recovering phosphorus and nitrogen from agricultural waste is precipitation. In precipitation processes, by means of a chemical reaction, the nutrients are recovered and separated by crystallisation. Depending on the reagents used in the process and the reaction carried out, crystal obtained as final product will be different. One of the products with the greatest projection is the struvite (an ammonium, phosphorus and magnesium salt) which can then be used as a slow-release biofertiliser (Le Corre et al. 2009). As can be seen in Eq. (1), struvite is obtained by reacting nitrogen (in the form of ammonium) and phosphorus (in the form of phosphate) contained in livestock waste, with a source of magnesium:

1 $$ {\mathrm{Mg}}^{2+}+{\mathrm{NH}}_4^{+}+{\mathrm{PO}}_4^{3-}+6{\mathrm{H}}_2\mathrm{O}\leftrightarrow {\mathrm{Mg}\mathrm{NH}}_4{\mathrm{PO}}_4\cdotp 6{\mathrm{H}}_2\mathrm{O} $$

An increase in the magnesium concentration and pH of the solution causes the equilibrium shift to struvite formation (Eq. (1)) and a decrease in struvite solubility. However, depending on the ions contained in the manure, a number of competitive reactions may also occur that would cause a decrease in struvite crystallisation yield (MgHPO_4_ (Eq. (2)), Mg(OH)_2_ (Eq. (3)) and CaHPO_4_ (Eq. (4)) (Mohan et al. 2011). According to Munir et al. (2017), the struvite precipitation reaction is usually the predominant one, as some of these competitive reactions are very slow, require pH values below 6 or are suppressed if a high concentration of Mg is present in the reaction medium (i.e. CaHPO_4_).

2 $$ {\mathrm{Mg}}^{2+}+\mathrm{H}{\mathrm{PO}}_4^{2-}\leftrightarrow \mathrm{MgH}{\mathrm{PO}}_4 $$

3 $$ {\mathrm{Mg}}^{2+}+2{\mathrm{OH}}^{-}\leftrightarrow \mathrm{Mg}{\left(\mathrm{OH}\right)}_2 $$

4 $$ {\mathrm{Ca}}^{2+}+{\mathrm{H}}^{+}+{\mathrm{PO}}_4^{3-}\leftrightarrow \mathrm{CaH}{\mathrm{PO}}_4 $$

Countries such as the Netherlands, Denmark, Belgium and Germany have already established special authorisations for the use of struvite as a fertiliser recovered from wastewater or manure. In many cases, the major impediment to phosphorus recovery in struvite form is because the solid fraction of livestock waste has an important part of phosphorus. According to Drosg et al. (2015), 55–65%w of the total amount of phosphorus from pig manure digestate is in the solid fraction. In order for this phosphorus to take part in the precipitation reaction, it should previously be recovered in the liquid fraction as soluble inorganic phosphorus. Therefore, it is necessary to carry out some pre-treatment to the crystallisation reaction of struvite to maximise phosphorus recovery.

These pre-treatments may consist of heat treatments (temperature modification), acid or alkaline treatments (pH modification) or use of chemical additives (addition of EDTA) (Latif et al. 2015). However, heat treatment presents high-energy costs and EDTA a high price (0.95 $/kg). Regarding alkaline treatments, according to previous studies (Bashir et al. 2019), a transformation of P species can be achieved, but the solubilisation and release of the nutrient will be small. Thus, tentatively, acid treatment would be the best alternative as a pre-treatment for the release of phosphorus (Zhang et al. 2010). Ottosen et al. (2013) reported an increase of 20–75% for phosphorus concentration in the liquid fraction, by subjecting dehydrated sludge to an acid treatment. Latif et al. (2015) subjected activated sludge to an acid treatment (pH lower than 5.7) obtaining 3.6 times more phosphorus release than under normal conditions (pH 7.7).

Therefore, the main objective of this work is to determine the feasibility of an acid pre-treatment, as a means of releasing the phosphorus contained in the solid of a livestock waste (digestate from the AD of pig manure), with the purpose of improving not only the technical performance of the phosphorus precipitation process but also its economic profitability (as struvite or calcium phosphate).

## Materials and methods

### Design of experiments

In order to carry out the tests of this work, a design of experiments (DOE) was made considering the most influential factors in the output variable under study (proportion of phosphorus released in the digestate liquid fraction). Since the technique selected for the phosphorus recovery has been an acid pre-treatment of the digestate, the factor that had the greatest influence on the output variable was the pH at which the acid pre-treatment has been carried out. pH of the digestate from the AD of pig manure is usually around 8.0; therefore, several pH levels, lower than the initial pH level, were selected: 7.0, 6.0, 5.0 and 4.0. On the other hand, it is important to evaluate the phosphorus recovery yield according to the waste fraction where the acid pre-treatment is applied. Thus, the pre-treatment was applied to the complete digestate (raw digestate), as well as to the digestate solid fraction and to the digestate liquid fraction. Finally, the experimental work was carried out from two different raw materials, with different storage periods. Therefore, another factor taken into account is the digestate storage time, considering fresh digestate (produce during the week) and old digestate (stored for 6 months) as process inputs.

Summarising, in the experimental study of this work, a DOE has been carried out considering the complete factorial of the three factors studied. As can be seen in Table 1, for the first factor (pH), five levels were considered, for the second (fraction of material), three levels and for the third factor (storage time), two levels. In Table 2, the experimental conditions for each test are presented. In the last part of this work, a study of the influence of the acid pre-treatment on the recovery of phosphorus by means of its precipitation as struvite (NH_4_MgPO_4_·6H_2_O) and calcium phosphate (Ca_3_(PO_4_)_2_) was carried out. For this purpose, a comparison of the phosphorus recovery yield was made for the two previous salts, considering and not considering acid pre-treatment (Table 3).

**Table 1:** DOE for phosphorus release study

Factors	Levels				
pH	4.0	5.0	6.0	7.0	8.0
Fraction of material	Raw digestate	Digestatesolid fraction		Digestate liquidfraction	
Storage age	Fresh digestate			Old digestate

**Table 2:** Operating conditions for each test

Test number	pH	Fraction of material	Storage age	Test number	pH	Fraction of material	Storage age
1.1.1	8.0	Raw digestate	Fresh digestate	4.1.1	8.0	Raw digestate	Old digestate
1.2.1	7.0	Raw digestate	Fresh digestate	4.2.1	7.0	Raw digestate	Old digestate
1.3.1	6.0	Raw digestate	Fresh digestate	4.3.1	6.0	Raw digestate	Old digestate
1.4.1	5.0	Raw digestate	Fresh digestate	4.4.1	5.0	Raw digestate	Old digestate
1.5.1	4.0	Raw digestate	Fresh digestate	4.5.1	4.0	Raw digestate	Old digestate
1.1.2	8.0	Raw digestate	Fresh digestate	4.1.2	8.0	Raw digestate	Old digestate
1.2.2	7.0	Raw digestate	Fresh digestate	4.2.2	7.0	Raw digestate	Old digestate
1.3.2	6.0	Raw digestate	Fresh digestate	4.3.2	6.0	Raw digestate	Old digestate
1.4.2	5.0	Raw digestate	Fresh digestate	4.4.2	5.0	Raw digestate	Old digestate
1.5.2	4.0	Raw digestate	Fresh digestate	4.5.2	4.0	Raw digestate	Old digestate
2.1.1	8.0	Solid fraction	Fresh digestate	5.1.1	8.0	Solid fraction	Old digestate
2.2.1	7.0	Solid fraction	Fresh digestate	5.2.1	7.0	Solid fraction	Old digestate
2.3.1	6.0	Solid fraction	Fresh digestate	5.3.1	6.0	Solid fraction	Old digestate
2.4.1	5.0	Solid fraction	Fresh digestate	5.4.1	5.0	Solid fraction	Old digestate
2.5.1	4.0	Solid fraction	Fresh digestate	5.5.1	4.0	Solid fraction	Old digestate
2.1.2	8.0	Solid fraction	Fresh digestate	5.1.2	8.0	Solid fraction	Old digestate
2.2.2	7.0	Solid fraction	Fresh digestate	5.2.2	7.0	Solid fraction	Old digestate
2.3.2	6.0	Solid fraction	Fresh digestate	5.3.2	6.0	Solid fraction	Old digestate
2.4.2	5.0	Solid fraction	Fresh digestate	5.4.2	5.0	Solid fraction	Old digestate
2.5.2	4.0	Solid fraction	Fresh digestate	5.5.2	4.0	Solid fraction	Old digestate
3.1.1	8.0	Liquid fraction	Fresh digestate	6.1.1	8.0	Liquid fraction	Old digestate
3.2.1	7.0	Liquid fraction	Fresh digestate	6.2.1	7.0	Liquid fraction	Old digestate
3.3.1	6.0	Liquid fraction	Fresh digestate	6.3.1	6.0	Liquid fraction	Old digestate
3.4.1	5.0	Liquid fraction	Fresh digestate	6.4.1	5.0	Liquid fraction	Old digestate
3.5.1	4.0	Liquid fraction	Fresh digestate	6.5.1	4.0	Liquid fraction	Old digestate
3.1.2	8.0	Liquid fraction	Fresh digestate	6.1.2	8.0	Liquid fraction	Old digestate
3.2.2	7.0	Liquid fraction	Fresh digestate	6.2.2	7.0	Liquid fraction	Old digestate
3.3.2	6.0	Liquid fraction	Fresh digestate	6.3.2	6.0	Liquid fraction	Old digestate
3.4.2	5.0	Liquid fraction	Fresh digestate	6.4.2	5.0	Liquid fraction	Old digestate
3.5.2	4.0	Liquid fraction	Fresh digestate	6.5.2	4.0	Liquid fraction	Old digestate

**Table 3:** Experiments on phosphorus recovery yield by precipitation

Factors	Levels	
Precipitation as struvite	With acidpre-treatment	No acidpre-treatment
Precipitation as calcium phosphate	With acidpre-treatment	No acidpre-treatment

### Methodology and experimental equipment

The experiments for the study of acid pre-treatment were carried out using batch stirred tank reactors of 250 mL volume. In each of the reactors, 100 mL of testing media was added, composed of the corresponding raw material (raw digestate, digestate solid fraction or digestate liquid fraction) and the required amount of sulphuric acid in each case. All tests were performed in duplicate, not taking into consideration and repeating the outliers. For acid hydrolysis, sulphuric acid was selected over other acids such as phosphoric or nitric, because there was a substantial difference in the cost: 200–220 €/t H_2_SO_4_ (Shijiazhuang Xinlongwei Chemical Co., Ltd. 2020), 620–700 €/t H_3_PO_4_ (Liuzhou Xianmi Trade Co., Ltd. 2020) and 290–330 €/t HNO_3_ (Langfang Jinhai Chemicals Industry Co., Ltd. 2020). Prior to experimentation, an initial characterisation of each digestate was performed (Table 4). The initial concentration of magnesium in the digestate samples is very small, so it is below the measurement range of the analysis equipment (ICP) and cannot be detected. The lower detection limit of ICP for magnesium is 1 mg/L.

**Table 4:** Initial characterisation for fresh digestate and old digestate

Parameter	Kind of digestate	
	Fresh digestate	Old digestate
[NH_4_^+^-N] initial (mg/L)	3863.52 ± 298.88	2181.85 ± 216.55
[P_T_-P] initial (mg/L)	2098.8 ± 16.49	181.12 ± 1.35
[Mg^2+^] initial (mg/L)	n.d.	n.d.
[Ca^2+^] initial (mg/L)	33.43 ± 1.44	14.12 ± 0.56
Solid fraction (%w)	38.29-40.69 ± 3.23	3.64-17.87 ± 1.42
pH	8.15-8.25 ± 0.1	8.04-8.05 ± 0.1

*n.d.* not detected

**Fig. 1:**
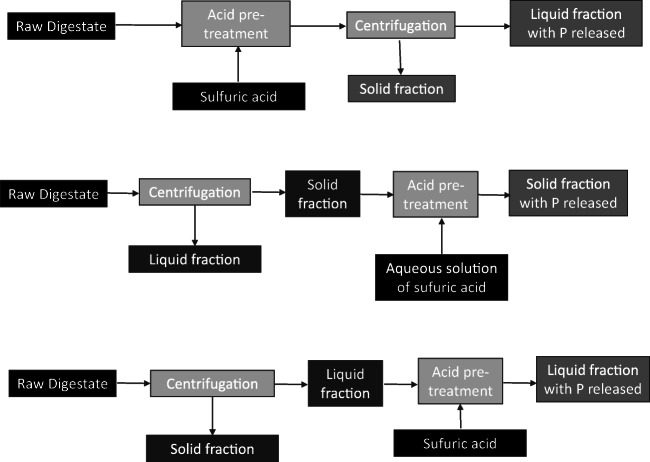
Diagram of the experimental procedure for acid pre-treatment

In the case of the raw digestate used as raw material, samples of 100 mL were introduced in the 250-mL batch reactors. To each of the reactors, the necessary quantity of sulphuric acid to reduce the pH level up to 7.0, 6.0, 5.0 or 4.0 was added. The samples were agitated (500 rpm) in a multi-position stirrer and allowed to react for 1 h. Separation was achieved by centrifugation at 5000 rpm for 10 min. Finally, the liquid fraction obtained from centrifugation was analysed to determine the amount of phosphorus present in the fraction. In tests with the digestate solid fraction, in the first step, the raw digestate was centrifuged for 10 min and 5000 rpm, the solid fraction was collected and dried for 48 h at 105 °C. Subsequently, in each 250-mL reactor, 1 g of dry solid fraction was dissolved with 100 mL of deionised water. The necessary amount of sulphuric acid was added to the aqueous solution in each case. Finally, the samples were left reacting for 1 h with 500 rpm agitation, in a multi-position stirrer. The solid and liquid fractions were separated by centrifugation (5000 rpm and 10 min) and the liquid fraction was analysed to determine its phosphorus concentration. To perform the digestate liquid fraction tests, the raw digestate was centrifuged for 10 min at 5000 rpm to separate the liquid and solid fractions. Each 250-mL batch stirred tank reactor was fed 100 mL of liquid fraction and the required portion of sulphuric acid. As in previous tests, the samples were agitated for 1 h at 500 rpm in a multi-position stirrer. Once the reaction was finished, the liquid fraction was recovered by centrifugation (5000 rpm and 10 min) and the concentration of phosphorus in this fraction was determined. The experimental conditions of the methods (temperature, centrifugation speed, reaction time) are original and have been adapted from previous studies (Corona et al. 2020). In all experiments, the amount of phosphorus recovered was determined, taking into account the concentration of phosphorus in the starting digestates and in the final liquid fraction of each experiment. A diagram of the experimental procedure carried out in each case is shown in Fig. 1.

Regarding the study of the influence of this acid pre-treatment on the phosphorus recovery by precipitation as struvite and calcium phosphate, the protocol is the following: In all cases, fresh digestate was used, the samples that were not subjected to acid pre-treatment had a pH of 8.0, approx., while the samples subjected to acid pre-treatment were added sulphuric acid until pH values of 7.0 and 5.0 were reached. On this occasion, the reaction volumes used were 50 mL. Once the samples with acid pre-treatment and the samples without acid pre-treatment were located in the corresponding reactor (batch stirred tank reactors), the reactions for the precipitation of struvite and calcium phosphate were carried out. In order to obtain struvite, a magnesium salt (MgCl_2_·6H_2_O) was added to the pre-treated and non-pre-treated samples in a molar ratio Mg/P = 1.5. In the case of calcium phosphate precipitation, calcium hydroxide (Ca(OH)_2_) was added to the pre-treated and non-pre-treated samples in a molar ratio Ca/P = 3.0. The N concentration in each of the samples remained practically constant, so the N/P ratio varied between 30 and 4, depending on the P concentration of each experiment. The solutions reacted for 1 h with a 500 rpm agitation in a multi-position stirrer. Finally, the struvite and calcium phosphate crystals, in each case, were separated from the reacting mixture by centrifugation (5000 rpm and 10 min). In the liquid fraction obtained in each of the experiments, the phosphorus concentration was analysed; thus, the percentage of phosphorus recovered by precipitation was determined, taking into account the initial concentration of phosphorus in each digestate. The pH values selected to test the effect of acid pre-treatment on phosphorus precipitation were the extremes of the range in which the optimum operating point would be found (between pH 5.0 and 7.0). To determine the optimum operating point, not only the acidification yield but also the Capital Expenditure (CAPEX) and Operational Expenditures (OPEX) of the process should be taken into account. The final pH at which the reactions took place was in the range of 9.0–9.5 for struvite and 7.0–9.0 for calcium phosphate. To increase the pH after acid pre-treatment, sodium hydroxide was added. The high values of pH and reaction temperature favour an increase in reaction yield, but also an increase in N loss in the form of NH_3_ gas (due to the shift in the balance of NH_4_^+^/NH_3_). Therefore, it is not recommended to work with reaction temperatures above 25–30 °C or pH values of 9.0–9.5 (El-Mashad et al. 2004; Huang et al. 2017).

### Analytical methods and instrumentation

Fresh digestate samples were obtained from an AD plant near Ghent (Belgium), while old digestate samples were collected from an AD plant in Almazan (Spain). Fresh digestate was stored at 4 °C in a refrigerator until it was used and old digestate was collected from the DA plant’s ponds and was stored for 6 months at room temperature until use. For the characterisation of the raw material, the concentration of ammonia nitrogen (NH_4_^+^-N), total phosphorus (P_T_-P), magnesium (Mg^2+^), calcium (Ca^2+^) and the pH was determined. Nitrogen was measured by titrimetric method using a distiller (Selecta, RAT 2), a digester (Selecta) and a digital burette (Bran). Total phosphorus was determined by vanadomolybdophosphoric acid spectrophotometry in a Shimadzu UV-VIS spectrophotometer, model UV-1603 and a Selecta digester, model RAT 2. Mg and Ca concentration was analysed with an inductively coupled plasma optical emission spectrophotometer (ICP-OES) (Shimadzu AA-6800, Japan). The analyses have been carried out following the current standard for water analysis in Spain (AENOR 2002; AENOR 2005; Apha A 2000). pH was determined by a potentiometric method using a Crison pH meter, model pH 25. Reagents used and struvite samples obtained were weighed using a Sartorius model TE 214S analytical balance. The dry solid fraction of the initial digestate was obtained by drying at 105 °C for 48 h using a Selecta Digitronic model stove. Separation of the liquid and solid fractions of the samples used in the experimentation was carried out by centrifugation at 5000 rpm for 10 min using a Jouan model B4i centrifuge. The reagents used in this work have been MgCl_2_·6H_2_O and Ca(OH)_2_ (Scharlau brand, pure grade). The characteristics and morphology of the crystals were obtained by a scanning electron microscope (SEM) analysis (FEI QUANTA 200). By means of X-ray diffraction (XRD), the qualitative identification of the mineralogical composition of the crystalline sample was carried out. A Bruker diffractometer model D8-Advance with Göebel mirror was used to carry out the analyses. Angle 2*θ* (diffraction angle) scans were collected from 5° to 75°, with a of 2*θ* step width of 0.05 and a sampling time of 3 s per step. The qualitative identification of the sample was done with the ICDD (International Center for Diffraction Data) database, being 01-071-2089, for the struvite.

## Results

### Acid pre-treatment study

The results obtained in the pre-treatment study for the different fractions of the fresh digestate are summarised in Table 5 and for the old digestate in Table 6.

**Table 5:** Phosphorus released for the various fractions of material from fresh digestate

Raw digestate				Solid fraction of digestate				Liquid fraction of digestate			
Test number	pH	P released (%)	Acid volume (mL)	Test number	pH	P released (%)	Acid volume (μL)	Test number	pH	P released (%)	Acid volume (mL)
1.1.1	8.15	12.51 ± 0.93	0.00	2.1.1	9.22	30.16 ± 1.46	0.00	3.1.1	7.95	---	---
1.2.1	7.35	15.82 ± 1.05	0.53	2.2.1	8.12	30.16 ± 1.94	3.00	3.2.1	7.54	89.86 ± 3.42	0.25
1.3.1	6.47	25.47 ± 1.50	2.00	2.3.1	5.33	39.03 ± 1.41	15.00	3.3.1	6.47	91.57 ± 3.17	1.00
1.4.1	4.67	88.85 ± 3.63	2.50	2.4.1	7.04	37.59 ± 1.01	5.00	3.4.1	5.72	91.85 ± 3.65	1.25
1.5.1	4.19	92.20 ± 4.83	2.70	2.5.1	3.94	51.45 ± 2.73	25.00	3.5.1	4.03	83.43 ± 3.80	1.35
1.1.2	8.25	13.79 ± 0.94	0.00	2.1.2	9.04	29.99 ± 2.02	0.00	3.1.2	8.17	---	---
1.2.2	7.56	11.62 ± 0.74	0.53	2.2.2	7.04	35.18 ± 2.05	5.00	3.2.2	7.06	92.57 ± 3.48	0.25
1.3.2	6.06	31.99 ± 1.96	2.00	2.3.2	5.97	40.46 ± 2.72	9.00	3.3.2	6.50	88.72 ± 3.11	1.00
1.4.2	5.46	83.65 ± 3.55	2.50	2.4.2	5.30	39.25 ± 2.15	15.00	3.4.2	5.66	85.79 ± 3.04	1.25
1.5.2	5.70	66.79 ± 2.86	2.70	2.5.2	4.38	50.44 ± 2.91	25.00	3.5.2	4.00	87.28 ± 3.79	1.35

**Table 6:** Phosphorus released for the various fractions of material from old digestate

Raw digestate				Solid fraction of digestate				Liquid fraction of digestate			
Test number	pH	P released (%)	Acid volume (mL)	Test number	pH	P released (%)	Acid volume (μL)	Test number	pH	P released (%)	Acid volume (mL)
4.1.1	8.05	14.16 ± 0.06	0.00	5.1.1	9.02	28.56 ± 0.92	0.00	6.1.1	7.91	---	---
4.2.1	7.03	16.60 ± 0.06	0.10	5.2.1	7.04	31.29 ± 1.44	5.00	6.2.1	6.93	98.40 ± 2.40	0.05
4.3.1	5.95	31.60 ± 0.23	0.40	5.3.1	5.99	36.38 ± 1.62	9.00	6.3.1	6.09	86.10 ± 3.77	0.45
4.4.1	5.01	90.87 ± 4.61	0.55	5.4.1	5.07	50.74 ± 1.69	16.00	6.4.1	5.11	79.15 ± 3.36	0.55
4.5.1	3.93	95.58 ± 4.18	0.65	5.5.1	3.94	54.01 ± 2.07	21.00	6.5.1	4.07	76.05 ± 4.06	0.65
4.1.2	8.04	12.30 ± 0.05	0.00	5.1.2	9.11	29.14 ± 1.30	0.00	6.1.2	8.23	---	---
4.2.2	7.07	18.46 ± 0.07	0.08	5.2.2	6.97	30.69 ± 1.34	4.00	6.2.2	7.06	81.50 ± 3.10	0.05
4.3.2	5.96	29.66 ± 0.96	0.55	5.3.2	6.06	37.21 ± 1.86	10.00	6.3.2	6.08	86.10 ± 4.42	0.40
4.4.2	5.08	86.58 ± 3.63	0.60	5.4.2	4.98	50.42 ± 1.94	1.00	6.4.2	4.92	83.00 ± 4.03	0.50
4.5.2	3.99	93.68 ± 4.07	0.65	5.5.2	4.01	52.95 ± 2.03	23.00	6.5.2	3.95	80.33 ± 3.93	0.65

**Fig. 2:**
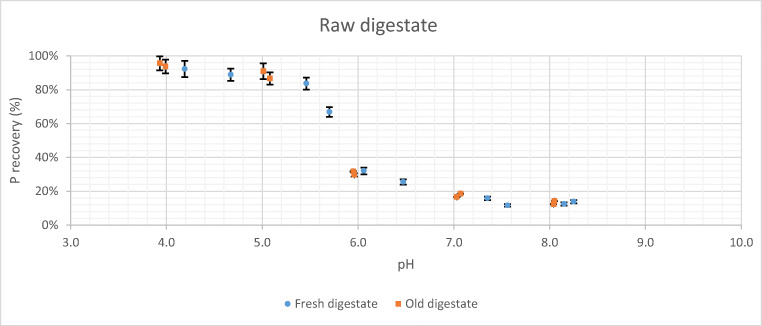
Phosphorus released as a function of pH and digestate age from raw digestate

In Fig. 2, the amount of phosphorus released into the liquid fraction as a function of pH (after acid pre-treatment) can be seen for the old and fresh digestate, when in both the addition of sulphuric acid in the raw digestate is performed. According to Fig. 2, the phosphorus percentage released from the raw digestate increases as the pH value decreases due to acid pre-treatment. The distribution of the experimental results presents a form of decreasing sigmoidal function, in which the inflection point is between the pH values of 5.0 and 6.0. Thus, from the initial pH value (around 8.0) to values of 6.0, the percentage of recovered phosphorus does not exceed 30%; however, for values close to pH 5.0, the recovery of phosphorus is around 90%. On the other hand, there is practically no difference between the results obtained for the fresh and old digestate, as far as the percentage of released phosphorus is concerned.

**Fig. 3:**
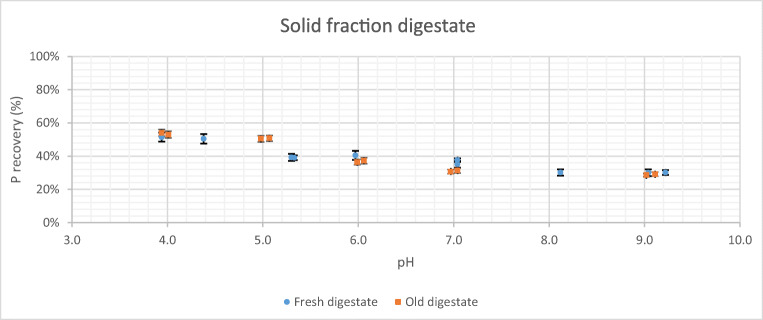
Phosphorus released as a function of pH and digestate age from solid fraction

In Fig. 3, the results of acid pre-treatment when acid is added to the solid fraction of both, fresh and old digestates, can be observed. In this case, the distribution of the results presents a more linear trend. Again, for high pH values (8.0–7.0) the recovery percentage is around 30–40% for all cases, but the percentage of phosphorus released for low pH values (4.0), i.e. when higher amounts of acid are added, does not reach 60%. Once more there are no noticeable differences between the results obtained for fresh digestate and old digestate.

**Fig. 4:**
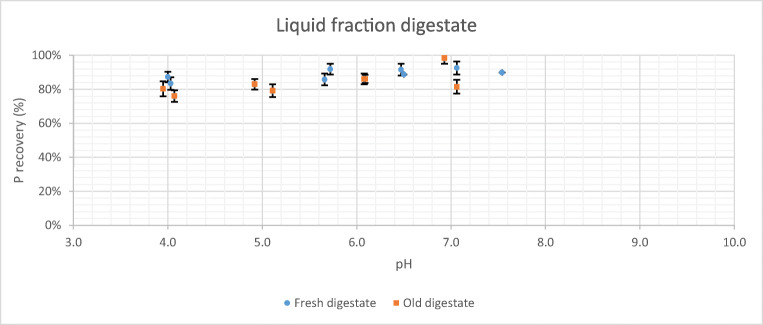
Phosphorus released as a function of pH and digestate age from liquid fraction

In Fig. 4, the results for the acid pre-treatment of liquid fraction from the fresh digestate and the old digestate are presented. In this case, the trend of the experimental results is practically a straight line of zero slope. Thus, there is hardly any difference between the pH at which the acid pre-treatment is performed and the percentage of phosphorus recovered. In addition, there is no difference in the results for fresh and old digestate.

### Study of the influence of acid pre-treatment on phosphorus recovery by precipitation of struvite and calcium phosphate

Table 7 presents the results of the experiments for the determination of the effect of acid pre-treatment on the precipitation of phosphorus both, in struvite and calcium phosphate form, using fresh digestate as raw material. In all cases, considerable improvements have been obtained when pre-treatment has been performed over when it has not been performed. This improvement is much more noticeable in the case of calcium phosphate, reaching phosphorus recovery percentages over 90% when acid pre-treatment is performed at pH 5.0. However, when acid pre-treatment is performed at pH 7.0, recovery is only 62%. In the case of struvite, the phosphorus recovery yield increases from 82 to 98% when pre-treatment is done at pH 5.0. This recovery only reaches 88% when pre-treatment is done at pH 7.0.

**Table 7:** Results of the experiments on the phosphorus recovery yield by precipitation from fresh digestate

Final product	Initial digestate fraction	pH initial	pH after pre-treatment	pH final	[NH_4_^+^-N] initial (mg/L)	[Mg^2+^] initial (mg/L)	[Ca^2+^] initial (mg/L)	[P] initial (mg/L)	[P] final (mg/L)	P recovered (%)	Acid volume (mL)	Alkali volume (mL)
Calcium phosphate	Liquid	8.11	---	8.24	3032.49 ± 290.36	n.d.	865.23 ± 7.78	222.96 ± 1.89	181.30 ± 1.66	18.68	---	---
Calcium phosphate	Whole	8.05	5.33	8.97	3218.27 ± 317.92	n.d.	6439.46 ± 60.93	1659.38 ± 11.36	52.95 ± 0.31	96.81	1.60	---
Struvite	Liquid	8.11	---	9.08	3032.49 ± 290.36	262.16 ± 1.39	26.38 ± 0.10	222.96 ± 1.89	38.96 ± 0.27	82.53	---	6.00
Struvite	Whole	8.05	5.33	9.43	3218.27 ± 317.92	1951.11 ± 14.20	27.11 ± 0.11	1659.38 ± 11.36	29.98 ± 0.23	98.19	1.60	7.00
Calcium phosphate	Liquid	8.19	---	8.19	2947.50 ± 287.44	n.d.	894.14 ± 8.01	230.41 ± 2.01	180.68 ± 1.63	21.58	---	---
Calcium phosphate	Whole	8.05	7.14	7.14	3169.81 ± 300.26	n.d.	1984.91 ± 12.54	511.49 ± 4.57	194.32 ± 1.71	62.01	0.40	---
Struvite	Liquid	8.16	---	8.95	2947.50 ± 287.44	270.92 ± 1.41	27.08 ± 0.11	230.41 ± 2.01	40.10 ± 0.28	82.60	---	6.00
Struvite	Whole	8.05	6.96	9.18	3169.81 ± 300.26	601.41 ± 3.24	25.75 ± 0.10	511.49 ± 4.57	57.55 ± 0.38	88.75	0.40	7.00

*n.d.* not detected

**Fig. 5:**
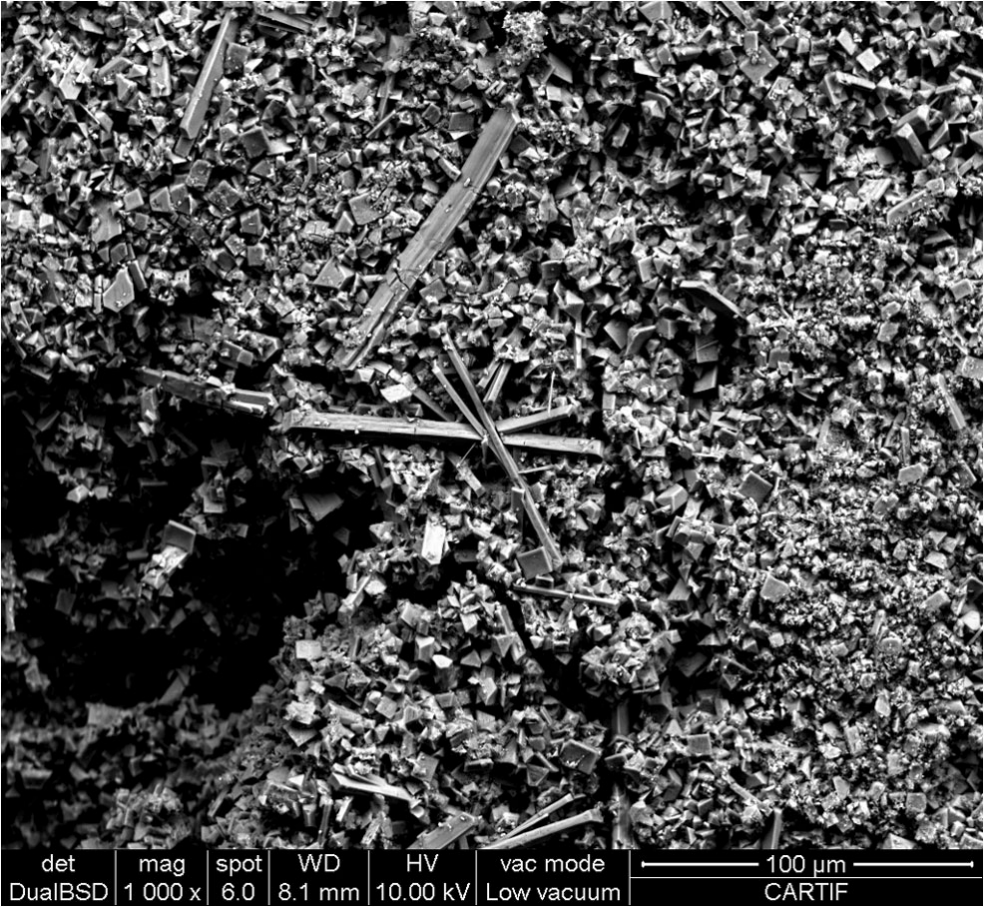
SEM picture of struvite obtained

**Fig. 6:**
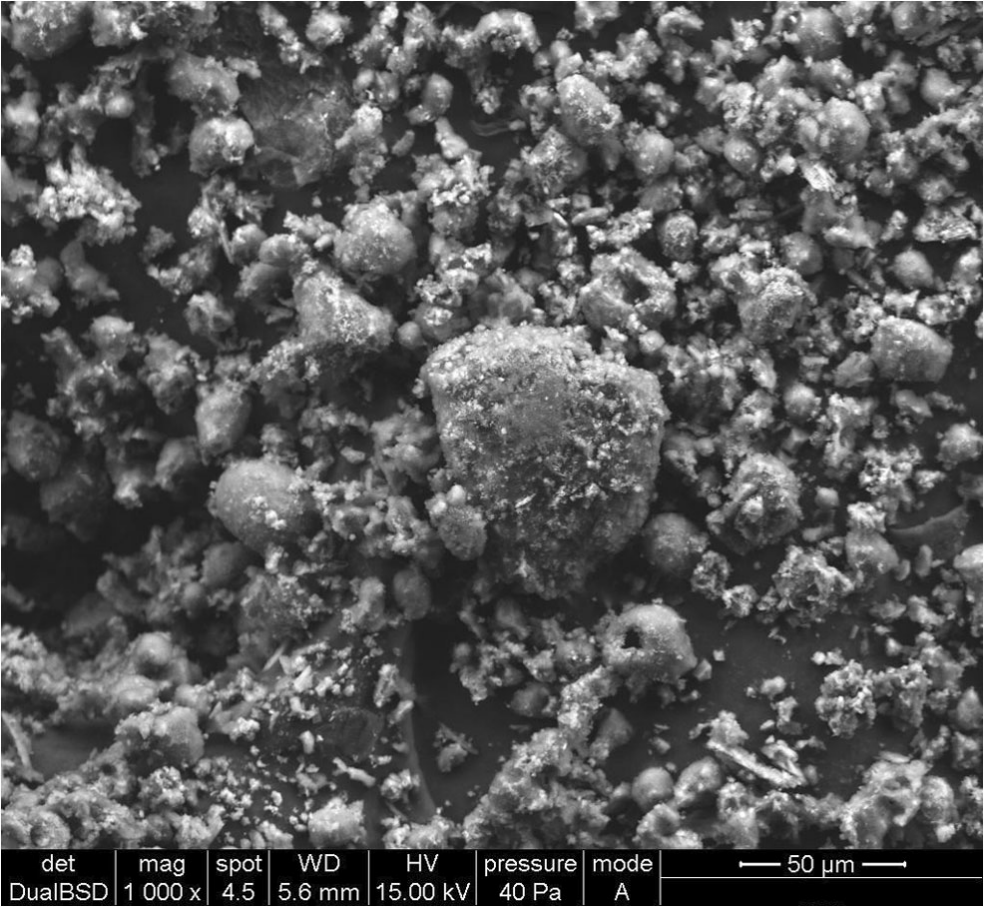
SEM picture of calcium phosphate obtained

Figure 5 and Fig. 6 show the struvite and calcium phosphate images obtained by SEM. In Fig. 5, characteristic needle shape of the struvite crystals can be observed, while in the case of Fig. 6, spherical-shaped particles indicate the typical morphology of the calcium phosphate.

**Fig. 7:**
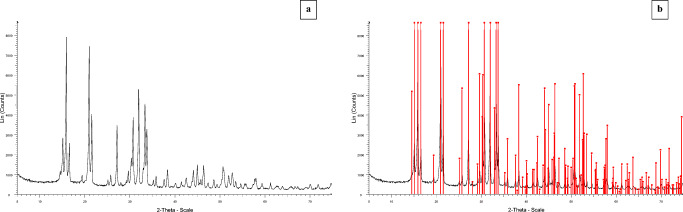
**a**, **b** XRD diagram from struvite obtained in the experiments

Figure 7a shows the XRD diagram for the struvite samples obtained in the experimental tests. The XRD diagram has been compared with a standard struvite XRD pattern (Fig. 7b) from the analytical equipment database library (01-071-2089). As shown in Fig. 7b, no noticeable differences in the position and intensity of the peaks can be seen when comparing the XRD diagram of the struvite obtained experimentally and from the library. Therefore, it can be admitted that the compound obtained as a product of the precipitation reaction is struvite.

## Discussion

According to the results obtained in this work, the technical feasibility of recovering more than 90% of the phosphorus contained in the digestate (from the AD of pig manure) by means of the acidification pre-treatment has been demonstrated. By this technique, it will be possible to increase the profitability of nutrient recovery processes, such as precipitation of phosphorus and nitrogen in the form of struvite or phosphorus in the form of calcium phosphate. This is because the digestate fraction used as raw material to obtain the above precipitates will have a higher concentration of available phosphorus if the acid pre-treatment is carried out; therefore, a greater amount of precipitate will be obtained with acidification process than without acid pre-treatment, for the same starting volume of digestate.

In concert with previous studies (Daumer et al. 2007; Shen et al. 2011; Schoumans et al. 2014; Zhang et al. 2010), phosphorus contained in the digestate solid fraction has been solubilised as inorganic phosphorus (mostly) in the liquid fraction of the digestate. Once the solid fraction was removed by centrifugation, the phosphorus available in the solid fraction could be recovered by chemical reaction (precipitation), either as struvite or as calcium phosphate (depending on the final target product).

The release of phosphorus from the digestate solid fraction and its solubilisation in the liquid fraction was achieved by lowering the pH of the digestate. This is explained by a solubility phenomenon, i.e. the solubility of phosphorus increases at low pH values. This drop in pH value was carried out by acid treatment. According to Schoumans et al. (2014) and other authors such as Daumer et al. (2010), the amount of acid that needs to be added varies in line with waste composition, initial pH of the waste and final pH that needs to be reached (4–25 kg H_2_SO_4_/t pig manure). As can be seen in Table 7, in the case of this study, the amounts of acid used are a bit higher (between 0.40 and 1.60 mL H_2_SO_4_/50 mL of digestate, that is, between 8 and 30 kg H_2_SO_4_/t pig manure). The main reason may be that, when handling small amounts of acid, the relative errors are much greater than if the operation were performed on a larger scale.

Even though acid treatment can be carried out by biological treatment instead of chemical treatment (inorganic acid), biological treatment was discarded because its operation times are much longer than those of chemical treatment. The chemical treatment is carried out in minutes while the biological treatment requires days of operation.

In this work, the amount of sulphuric acid added to lower the pH value of the digestate has been greater than that indicated by the digestate neutralisation reaction. This is due to the fact that the drop in pH is affected by intrinsic factors of the digestate, such as buffer capacity (this in turn depends on the concentration of bicarbonates and ammonium in the digestate). In addition, age of the digestate can also be another important factor, since the old manure will have a lower organic load (due to decomposition), which will lead to a higher concentration of ammonium and bicarbonates, causing greater digestate buffering capacity. However, no notable differences have been found in this work between the experiments carried out for the old digestate and the fresh digestate. The plausible explanation is that the composition of the initial mixture in each case is different.

Although acid treatment has been used successfully by other authors for phosphorus release and dewatering activated sludge (Antakyali et al. 2013; Cai et al. 2018), there is not much information on its application to digestate from livestock waste. Nevertheless, in this work, as far as the acid treatment for raw digestate is concerned, the results obtained are very encouraging, since it is possible to recover amounts of phosphorus higher than 90%, for pH values close to 4.0 (Fig. 2). However, it is necessary to point out that possibly the optimum operating point (both technical and economic) of this pre-treatment is for pH values between 5.0 and 6.0 (Fig. 2), as more than 80% of the phosphorus is released in this range. Working at a pH higher than 4.0 will result in less aggressive operating conditions for the equipment and considerably lower quantities of acid used. These results are in line with those obtained in previous works (Latif et al. 2015; Lundehøj et al. 2019; Schoumans et al. 2014), or even improve the results obtained by Bi et al. (2012) for the release of phosphorus from waste activated sludge (25%).

Phosphorus is usually present in the digestate in its soluble form (liquid fraction) and in the solid fraction particles. Therefore, it was necessary to carry out a study of the treatment to the solid fraction. Nevertheless, the acid treatment of the digestate solid fraction was not as successful as the treatment for the raw digestate. In this case, only phosphorus recovery around 50–60% was achieved for pH values between 4.0 and 5.0 (Fig. 3). According to Schoumans et al. (2014), this may be due to most of the phosphorus content in the solid fraction is usually found in very small particle sizes (51% is found in particles < 100 μm), so that these particles may have been incorporated into the liquid fraction when carrying out the previous separation by centrifugation (Fig. 1). On the other hand, the low yield for phosphorus release, in this case, may also be because acid treatment is less effective when it acts directly on the solid particles than on the dissolved particles, since acid has a greater impediment to accessing the phosphorus molecules in the undissolved solid and being able to release them.

When acid treatment is carried out on the liquid fraction, the results obtained in terms of phosphorus release are very similar (between 80 and 90%) for all pH values. This is due to the release of acid in soluble form is very similar in all cases and the differences between tests may be because the liquid fraction contains more or less small particles that can make an extra contribution of phosphorus. All this is in accordance with what Tasistro et al. (2007) or Szogi and Vanotti (2009) have reported.

With regard to the amount of acid added, it is necessary to bear in mind that it can represent a fundamental part of operating costs when the addition of acid is used as a pre-treatment system in some phosphorus recovery technology (such as precipitation). Therefore, the selection of the pre-treatment operating conditions can be a determining factor not only for the technical but also economic viability of the process. Taking into account the price of chemicals, acid pre-treatment can represent up to 25% of the operating costs of the phosphorus recovery process by precipitation (Schoumans et al. 2014; Schröder et al. 2009). A priori the best option from the economic point of view would be to carry out the acid pre-treatment to the solid fraction, since this is the option in which the consumption of acid is lower. However, the yield obtained is much lower than the pre-treatment to the raw digestate or liquid fraction. Thus, combining technical and economic performance, the best options for acid pre-treatment would be the last two (raw digestate or liquid fraction). Between the two options, the results are more favourable for the raw digestate, since, although the phosphorus release yields are similar, the amount of phosphorus recovered for the raw digestate is much higher, which will lead to an increase in economic yield.

Finally, regarding the phosphorus precipitation process, as can be seen in Table 7, the best results obtained in terms of reaction yield (percentage of phosphorus recovered) and the amount of phosphorus precipitated are those in which acid pre-treatment is included, both for struvite and calcium phosphate precipitation. There are substantial differences when acid pre-treatment is performed at pH 7.0 or pH close to 5.0. Possibly, the technical and economic optimum for both struvite and calcium phosphate is at a pH value around 6.0 for acid pre-treatment. To encourage P precipitation in the form of struvite or calcium phosphate, it is necessary to increase the supersaturation of the solution. This is achieved either by increasing pH value or concentration of the reacting agents (N, P, Mg or Ca), especially P, since in both cases it is the limiting reagent. Most studies of struvite precipitation are conducted between pH values of 8.0–10.5 (Stolzenburg et al. 2015; Li et al. 2019) and Mg/P ratios of 1.0–2.0 (Kumar and Pal 2015). In the present study, struvite P recovery tests have been carried out at pH values of 9.0–9.5 and Mg/P ratio of 1.5, obtaining P recovery yields between 82 and 98%. These yields are similar or even higher than obtained by other authors under similar conditions using pig slurry as raw material without pre-treatment. Corona et al. (2020) obtained P recovery yields of 62% under the same conditions, using batch stirred tank reactors of 500 mL volume. However, authors such as Li et al. (2012) or Tang et al. (2018) conclude that a Mg/P ratio between 1.0 and 2.0 increases the degree of supersaturation and significantly favours the reaction performance. Zhou et al. (2015) obtained an increase in P removal from 29% for Mg/P = 0.2 to 91% for Mg/P = 1.5. These results are also consistent with those obtained by Barbosa et al. (2016). As demonstrated by authors such as Capdevielle et al. (2013), as Mg/P ratio increases, P recovery yield is higher, since the struvite saturation index is proportional to the logarithm of the concentrations of the crystal reacting species (PO_4_^3−^, Mg_2_^+^ and NH_4_^+^). Furthermore, this yield was kept practically constant for values higher than Mg/P = 1.5. On the other hand, Huang et al. (2016) and Shih et al. (2017) confirmed that it is not advisable to work with pH values higher than 10.5, since there is a significant loss of N in gaseous form (NH_3_), due to the fact that the reaction balance NH_4_^+^/NH_3_ moves towards the gas form for high pH values.

However, to corroborate the beneficial effect of acid pre-treatment on the P recovery, it would be necessary to carry out a pilot scale study in which the results obtained are closer to the industrial scale. In that study, the main factors that should be taken into account to establish the optimum operating conditions would be the amount and cost of chemical reagents used (both in the acid pre-treatment process and in the precipitation process), as well as the amount and sale price of the final products obtained (struvite or calcium phosphate).

It is clear that the economic impact of including an acid pre-treatment in the different phosphorus recovery options by precipitation should be analysed on a larger scale (pilot plant). Nevertheless, from the results obtained in this work, it is estimated that the operating costs of acid pre-treatment (mostly represented by the cost of chemicals) can vary from a few euro cents per cubic metre of digestate treated (around 0.30 €/m^3^) to 6 €/m^3^ digestate treated. This variability is influenced by the characteristics of the digestate to be treated, mainly due to three main parameters: phosphorus concentration, amount of solid fraction and size of the solid particles present in the digestate. Obviously, this cost difference represents an important factor that will determine the viability of the nutrient recovery process.

To conclude, it is necessary to point out that, once the phosphorus has been recovered in the form of struvite, a digestate with a much lower phosphorus and nitrogen content is obtained as by-product of the process. This digestate with low nutrient content will be able to be used directly on the land and the crop by fertigation, as it will comply with current legislation regarding its nitrogen and phosphorus content. All this will mean an extra benefit, which will result in greater profitability of the process.

## Conclusions

In the present work, an experimental procedure has been carried out to determine the potential of acid pre-treatment, as a technique to recover the phosphorus contained in the solid phase of the digestate. A design of experiments was achieved to investigate the influence of the main parameters, determining the acid pre-treatment, such as pH, the fraction of digestate to be treated and the age of the digestate. According to the results obtained, the pH is the most influential factor in the acid pre-treatment for the release of phosphorus from the solid to the liquid fraction of the digestate. The optimal pH values for pre-treatment are between 6.0 and 5.0. On the other hand, the application of the acid pre-treatment directly to the raw digestate is considered the best option as higher phosphorus concentrations are released by this alternative. In addition to the acid pre-treatment study itself, the effect of acid pre-treatment on phosphorus precipitation in the form of struvite and calcium phosphate was investigated. The efficiency of the phosphorus precipitation reaction was increased up to 15% for struvite crystallisation and by 80% for calcium phosphate by the acid pre-treatment. For both struvite and calcium phosphate precipitation, the amount of phosphorus available in the liquid fraction of the digestate was increased by 7.5 times through acid pre-treatment (from 230.41 to 1659.39 mgP/L).

## Data Availability

The datasets used and/or analysed during the current study are available from the corresponding author on reasonable request.
