# Correction to: Study of pig manure digestate pre-treatment for subsequent valorisation by struvite

**DOI:** 10.1007/s11356-022-21026-y

**Published:** 2022-05-25

**Authors:** Francisco Corona, Dolores Hidalgo, Jesús María Martín-Marroquín, Erik Meers

**Affiliations:** 1 CARTIF Centro Tecnológico, 47151 Boecillo Valladolid, Spain; 2 ITAP Institute, University of Valladolid, 47010 Valladolid, Spain; 3 Department of Applied Analytical and Physical Chemistry, Faculty of Bioscience Engineering, Ghent University, Coupure Links 653, 9000 Ghent, Belgium

**Correction to: Environmental Science and Pollution Research (2021) 28:24731–24743**

**https://doi.org/10.1007/s11356-020-10918-6**

The article Study of pig manure digestate pre-treatment for subsequent valorisation by struvite written by Francisco Corona, Dolores Hidalgo, Jesús María Martín-Marroquín and Erik Meers, was originally published electronically on the publisher’s internet portal on Oct 3 2020 without open access. With the author(s)’ decision to opt for Open Choice the copyright of the article changed on 18 May 2022 to © The Author(s) 2021 and the article is forthwith distributed under a Creative Commons Attribution 4.0 International License, which permits use, sharing, adaptation, distribution and reproduction in any medium or format, as long as you give appropriate credit to the original author(s) and the source, provide a link to the Creative Commons licence, and indicate if changes were made. The images or other third party material in this article are included in the article’s Creative Commons licence, unless indicated otherwise in a credit line to the material. If material is not included in the article’s Creative Commons licence and your intended use is not permitted by statutory regulation or exceeds the permitted use, you will need to obtain permission directly from the copyright holder. To view a copy of this licence, visit http://creativecommons.org/licenses/by/4.0.

The Original article has been corrected.

